# Increasing plasma calprotectin (S100A8/A9) is associated with 12-month mortality and unfavourable functional outcome in critically ill COVID-19 patients

**DOI:** 10.1186/s40560-024-00740-4

**Published:** 2024-07-09

**Authors:** Ingrid Didriksson, Maria Lengquist, Martin Spångfors, Märta Leffler, Theodor Sievert, Gisela Lilja, Attila Frigyesi, Hans Friberg, Alexandru Schiopu

**Affiliations:** 1 Department of Clinical Sciences, Anaesthesiology and Intensive Care, Lund University, Lund, Sweden; 2 Intensive and Perioperative Care Unit, Skåne University Hospital, Malmö, Sweden; 3 Intensive and Perioperative Care Unit, Skåne University Hospital, Lund, Sweden; 4 Anaesthesia, and Intensive Care Unit, Kristianstad Hospital, Kristianstad, Sweden; 5 Department of Neurology, Skåne University Hospital, Lund, Sweden; 6 Department of Translational Medicine, Lund University, Malmö, Sweden; 7 Department of Internal Medicine, Skåne University Hospital, Lund, Sweden; 8 Nicolae Simionescu Institute of Cellular Biology and Pathology, Bucharest, Romania

**Keywords:** COVID-19, ICU, Calprotectin, Innate immune system, Mortality

## Abstract

**Background:**

Calprotectin (S100A8/A9) is a pro-inflammatory mediator primarily released from neutrophils. Previous studies have revealed associations between plasma calprotectin, disease severity and in-hospital mortality in unselected COVID-19 patients.

**Objective:**

We aimed to assess whether plasma calprotectin dynamics during the first week of intensive care are associated with mortality and functional outcome in critically ill COVID-19 patients.

**Methods:**

This prospective study included 498 COVID-19 patients admitted to six intensive care units (ICUs) in Sweden between May 2020 and May 2021. Blood samples were collected on ICU admission and on day 7. The primary outcome was 12-month mortality. Secondary outcomes were functional outcome of survivors at 3 and 12 months, and the need for invasive mechanical ventilation (IMV) or continuous renal replacement therapy (CRRT) during the ICU stay. Functional outcome was assessed by the Glasgow Outcome Scale Extended (GOSE, range 1–8, with < 5 representing an unfavourable outcome). Associations between plasma calprotectin and outcomes were examined in binary logistic regression analyses adjusted for age, sex, BMI, hypertension, smoking, and creatinine.

**Results:**

High plasma calprotectin on admission and day 7 was independently associated with increased 12-month mortality. Increasing calprotectin from admission to day 7 was independently associated with higher mortality at 12 months [OR 2.10 (95% CI 1.18–3.74), *p* = 0.012], unfavourable functional outcome at 3 months [OR 2.53 (95% CI 1.07–6.10), *p* = 0.036], and the use of IMV [OR 2.23 (95% CI 1.10–4.53), *p* = 0.027)] and CRRT [OR 2.07 (95% CI 1.07–4.00), *p* = 0.031)]. A receiver operator characteristic (ROC) model including day 7 calprotectin and age was a good predictor of 12-month mortality [AUC 0.79 (95% CI 0.74–0.84), *p* < 0.001]. Day 7 calprotectin alone predicted an unfavourable functional outcome at 3 months [AUC 0.67 (95% CI 0.58–0.76), *p* < 0.001].

**Conclusion:**

In critically ill COVID-19 patients, increasing calprotectin levels after admission to the ICU are associated with 12-month mortality and unfavourable functional outcome in survivors. Monitoring plasma calprotectin dynamics in the ICU may be considered to evaluate prognosis in critical COVID-19.

*Study registration: ClinicalTrials.gov Identifier*: NCT04974775, registered April 28, 2020.

**Supplementary Information:**

The online version contains supplementary material available at 10.1186/s40560-024-00740-4.

## Introduction

COVID-19, caused by severe acute respiratory syndrome coronavirus 2 (SARS-CoV-2), is a highly inflammatory disease with varied clinical courses, from mild cases to critical disease [1]. Critical COVID-19 has been associated with immune system dysregulation, characterised by a cytokine storm, increased counts of circulating neutrophils, and lymphopenia [2–4]. This exaggerated inflammatory response may lead to severe respiratory distress, multiple organ failure and death [5].

Calprotectin is a heterodimer of two calcium-binding protein subunits, S100A8 and S100A9, and is an important pro-inflammatory innate immune mediator. Calprotectin is abundant in the cytoplasm of neutrophils and is also found at lower levels in monocytes, platelets, and epithelial cells [6]. Neutrophil activation releases large amounts of calprotectin in the extracellular space. Calprotectin induces nuclear factor kappa-light-chain-enhancer of activated B cells (NF-κB) and NOD-like receptor pyrin-domain-containing 3 (NLRP3) inflammasome activation in immune cells, leading to the production of inflammatory cytokines and chemokines. Moreover, calprotectin has been identified as an important chemoattractant for neutrophil migration to inflammatory sites [7]. The increased extravasation of neutrophils into the tissues in severe infections makes blood neutrophil count an unreliable marker for the intensity of the innate immune response in sepsis. Calprotectin is a stable protein with relatively slow elimination that may serve as a more reliable biomarker for the innate immune response in sepsis, reflecting the activation of neutrophils and other myeloid cells [8, 9].

Inflammatory biomarkers such as C-reactive protein (CRP), neutrophil-to-lymphocyte ratio (NLR), and interleukin-6 (IL-6) have been reported to be associated with the clinical course of COVID-19 during hospitalisation [10–12]. However, reliable predictors of mortality and long-term functional outcome in COVID-19 survivors are missing. Recent studies, including meta-analyses, suggest that high calprotectin levels correlate with COVID-19 disease severity [2, 13] and can predict intensive care unit (ICU) admission in unselected patient cohorts [14–17]. The ability of calprotectin to reflect long-term patient prognosis has not been examined.

This study aimed to assess whether the dynamics of plasma calprotectin during the first week of ICU care are associated with long-term mortality, functional outcome, and disease progression in critically ill COVID-19 patients.

## Material and methods

### Study design and data collection

This prospective multicentre cohort study included critically ill adult patients (≥ 18 years old) with laboratory-confirmed SARS-CoV-2 infection at six ICUs in the Skåne region, Sweden, between May 11th 2020 and May 10th 2021 [18]. We held weekly online meetings to ensure that similar admission and discharge criteria, as well as similar treatment protocols were used across all centres.

The study had a population-based design, predetermined to close after one year. Patients were excluded if COVID-19 was not the primary cause of ICU admission. Information on clinical patient characteristics such as age, body mass index (BMI), smoking status, Sequential Organ Failure Assessment (SOFA) [19], Simplified Acute Physiology Score 3 (SAPS 3) [20], Clinical Frailty Scale (CFS) [21], and Charlson Comorbidity Index (CCI) [22], was collected during the ICU stay. Glasgow Outcome Scale Extended (GOSE) [23] was assessed at 3 and 12-month follow-ups primarily performed face-to-face, with the option of interviewing via telephone as previously described [18]. Smoking was defined as active smoking or a history of smoking. Written informed consent was collected from all participants on admission, before discharge, or at a follow-up visit up to one year later, with a possibility for the participants to opt out at any given time. Consent was presumed for deceased patients. The Swedish Ethical Review Authority approved the SWECRIT COVID-19 study (Dnr: 2020-01955, 2020-03483, 2021-00655).

### Blood sample collection and calprotectin measurement

Blood samples for calprotectin measurement were collected on ICU admission and on day 7. All samples were centrifuged, aliquoted, and stored at − 80 °C in the SWECRIT COVID-19 biobank hosted within the central biobank of Region Skåne, Sweden (BD-47). Plasma calprotectin was analysed after the completion of the study at a certified clinical chemistry laboratory at Uppsala University Hospital, Sweden. A particle-enhanced turbidimetric assay (PETIA) was used with calprotectin-specific reagents supplied by Gentian AS (Moss, Norway) in a Mindray BS380 chemistry analyser (Mindray, Shenzhen, China) [24]. The method generates results in ~ 10 min.

### Outcomes

The study's primary outcome was 12-month mortality. The secondary outcomes were the functional outcome of survivors at 3 and 12 months, assessed by the clinician-reported Glasgow Outcome Scale Extended (GOSE), and the development of in-hospital organ failure, reflected by the need for invasive mechanical ventilation (IMV) or continuous renal replacement therapy (CRRT). GOSE is an ordinal scale that ranges from 1 to 8. In this scale, GOSE 1 represents death, GOSE 2 represents a vegetative state, GOSE 3–4 represents a patient with a severe disability who is dependent on daily support, GOSE 5–6 represents a patient with a moderate disability but is independent in daily life, GOSE 7 represents good recovery, but with minor disabilities, and GOSE 8 represents full recovery. Unfavourable functional outcome was defined as GOSE < 5, which reflects a person dependent on help in their daily life. To increase the inter-observer reliability of the GOSE scoring, all outcome assessors underwent mandatory training and were provided with a written manual. Certified interpreters were used for participants who were not fluent in Swedish.

### Statistics

Continuous variables are presented as median and interquartile range (IQR). Categorical variables are expressed as numbers and percentages (%). The Chi-square test was used to compare categorical variables between the two groups. Differences in continuous variables between the two groups were compared using the Mann–Whitney *U* test.

Plasma calprotectin values were normalised by log10 transformation and expressed as *Z*-scores before being used in the regression analyses. To assess calprotectin dynamics, we classified participants based on whether their calprotectin values increased or decreased from ICU admission to day 7. We performed binary logistic regression and Cox proportional hazard analyses to explore the association between calprotectin and mortality, functional outcome, and in-hospital outcomes. We used an unadjusted model (Model 1) and a model adjusted for the potential confounders age, sex, BMI, hypertension, smoking status, and creatinine (Model 2). Concerns have been raised about the potential interference of CRRT with plasma calprotectin levels in critical care [25]. Therefore, a sensitivity analysis was performed for calprotectin on day 7, excluding patients with CRRT.

To assess the ability of calprotectin to provide long-term prognostic information in our cohort of critically ill COVID-19 patients, we used receiver operator characteristic (ROC) curves with c-statistics. To create predictive models, we initially performed a multivariable binary logistic regression analysis with backward factor selection using 12-month mortality as the outcome and a *p*-value < 0.01, to identify the strongest independent predictors. We included age, sex, BMI, hypertension, smoking, creatinine, and calprotectin as covariates. Day 0 and day 7 calprotectin were included in separate models. In ROC curves, we investigated the discriminative ability of calprotectin to predict 12-month mortality and unfavourable functional outcome, alone or in combination with age. For day 7 calprotectin, we only included patients alive at ICU day 7. We used the DeLong test [26] to compare the different prediction models' areas under the ROC curve (AUC). The Youden index [27] was employed to select the threshold value for calprotectin with the optimal sensitivity/specificity balance for outcome prediction. All statistical analyses were performed using the Statistical Package for the Social Sciences (SPSS) version 27. A *p*-value < 0.05 was considered statistically significant.

## Results

### Clinical characteristics of the cohort

**Fig. 1:**
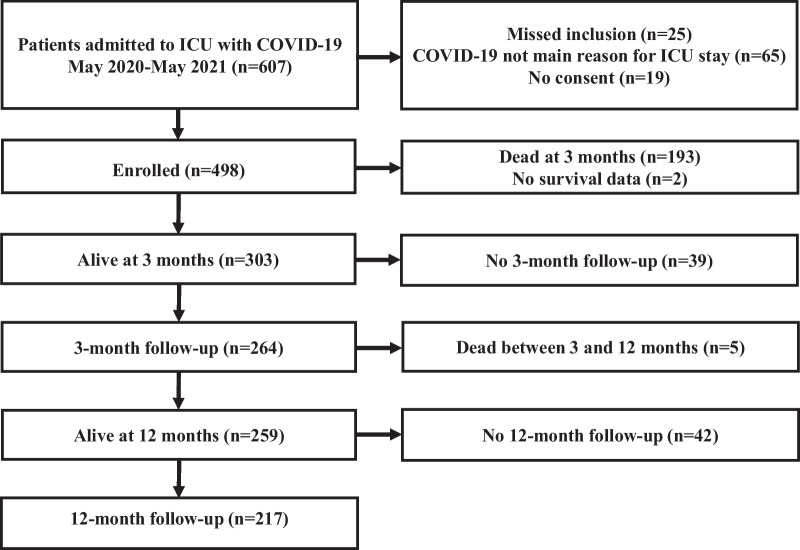
Flowchart of the study population

The study included 498 patients (Fig. 1). Mortality was 40% (*n* = 198) at 12 months. Invasive mechanical ventilation (IMV) was used in 72% (*n* = 357) of the patients, and 15% (*n* = 74) required CRRT. The use of IMV was significantly higher in non-survivors, whereas the use of CRRT was similar between groups (Table 1). At 3 months, 264 of 303 (87%), and at 12 months, 217/298 (73%) survivors participated in a follow-up. An unfavourable functional outcome (GOSE < 5) was found in 59/260 (23%) of survivors with complete GOSE data at 3 months and in 16/215 (7%) at 12 months (Supplementary Figs. 1 and 2). No participants were in a vegetative state (GOSE 2).

**Table 1:** Clinical characteristics at baseline stratified by 12-month mortality

	All patients	Survivors	Non-survivors	*p*-value
	*N* = 498	*N* = 298	*N* = 198	
Age (years)	66 [56–73]	61 [52–68]	72 [66–77]	< 0.001
Male sex	367 (74%)	218 (73%)	147 (74%)	0.79
BMI (kg/m^2^)	30 [27–35]	31 [27–36]	29 [26–33]	< 0.001
Smoking	220 (44%)	109 (38%)	111 (56%)	< 0.001
Diabetes mellitus	154 (31%)	85 (28%)	68 (34%)	0.17
Hypertension	270 (55%)	149 (51%)	121 (62%)	0.023
Symptomatic days before ICU	11 [8–15]	10 [7–14]	12 [9–16]	0.022
COPD or severe asthma	94 (19%)	51 (17%)	43 (22%)	0.20
Clinical Frailty Scale	3 [2–4]	3 [2, 3]	3 [2–4]	< 0.001
Charlson Comorbidity Index	3 [2–4]	2 [1–3]	4 [3–5]	< 0.001
SOFA	8 [5–9]	7 [4–9]	8 [5–10]	0.043
SAPS 3	60 [50–69]	56 [47–66]	66 [56–75]	< 0.001
*Laboratory findings*				
Creatinine (μmol/L)	75 [60–102]	70[58–93]	86 [65–125]	< 0.001
Calprotectin day 0 (mg/L) (*n* = 484)	7.39 [4.36–11.5]	6.72 [4.01–10.4]	8.62 [4.99–14.4]	< 0.001
Calprotectin day 7 (mg/L) (*n* = 356)	4.13 [2.30–7.01]	3.52[1.99–62]	6.01 [3.02–10.8]	< 0.001
*Clinical parameters*				
P/F ratio (min) (kPa)^a^	10 [8.0–12]	10 [8.0–13]	9 [7.0–14]	0.037
IMV	357 (72%)	196 (66%)	160 (81%)	< 0.001
CRRT	74 (15%)	45 (15%)	29 (15%)	0.89
IMV total days (*n* = 357)^a^	9 [5–18]	8 [5–17]	13 [7–21]	0.001
LOS ICU (*n* = 498)	10 [5–17]	9 [5–18]	10 [5–16]	0.93
LOS Hospital (*n* = 498)	23 [15–42]	23 [14–44]	22 [15–37]	0.95

Results are expressed as *n* (%) or median [IQR]

*BMI* body mass index, *ICU* intensive care unit, *COPD* chronic obstructive pulmonary disease, *SOFA* Sequential Organ Failure Assessment, *SAPS* *3* Simplified Acute Physiology Score, P/F ratio defined as P_a_O_2_ (arterial partial pressure of oxygen)/F_i_O_2_ (fraction of inspired oxygen), *IMV *invasive mechanical ventilation, *CRRT* continuous renal replacement therapy, *LOS* length of stay, *ICU* intensive care unit

^a^Lowest P/F ratio on ICU day 1

### Calprotectin levels are elevated in patients with unfavourable outcomes

Plasma samples were available for 484 of 498 participants on admission and 356 on day 7 (Table 1). Missing samples on day 7 were due to death before day 7 (*n* = 56), ICU discharge to other units and hospital wards (*n* = 51) and missed blood sampling (*n* = 35). Calprotectin levels on ICU admission and day 7 were significantly higher in non-survivors (Table 1). Patients with unfavourable functional outcome at 3 months had significantly higher calprotectin levels on day 7 and increasing levels from admission to day 7 (Supplementary Table 1 and Supplementary Fig. 3).

### Associations between plasma calprotectin and outcomes

In unadjusted binary logistic regression models, elevated calprotectin on admission and day 7 was associated with increased 12-month mortality (Table 2). The associations remained significant after adjusting for age, sex, BMI, hypertension, smoking status, and creatinine (Table 2). Elevated day-7 calprotectin was independently associated with an unfavourable functional outcome (GOSE < 5) at 3 months among survivors (Table 3). Regression models for unfavourable functional outcomes at 12 months could not be adequately fitted due to the small percentage of patients with a GOSE score < 5 at this time point (Supplementary Fig. 2). In a sensitivity analysis excluding patients with CRRT, the association between day-7 calprotectin, mortality, and functional outcome remained significant (Supplementary Table 2).

**Table 2:** Associations between plasma calprotectin and mortality

	Model	12-month mortality		
		OR^a,b^	CI	*p*
Calprotectin day 0	1	1.37	1.13–1.65	0.001
	2	1.51	1.20–1.90	< 0.001
Calprotectin day 7	1	2.3	1.75–3.02	< 0.001
	2	1.89	1.34–2.68	< 0.001
Increasing calprotectin day 0–day 7	1	2.13	1.28–3.53	0.004
	2	2.10	1.18–3.74	0.012

Binary logistic regression model

Adjustment models: Model 1: unadjusted. Model 2: adjusted for age, sex, BMI, hypertension, smoking and creatinine

*GOSE* Glasgow Outcome Scale Extended

^a^Odds ratio (OR) expressed per 1 standard deviation (SD) increase in calprotectin

^b^OR for outcomes in patients with increasing calprotectin levels from day 0 to day 7

**Table 3:** Associations between plasma calprotectin and functional outcome

	Model	3-month GOSE < 5 (*n* = 59)		
		OR^ab^	CI	*p*
Calprotectin day 0	1	1.01	0.75–1.38	0.93
	2	0.98	0.70–1.36	0.88
Calprotectin day 7	1	1.94	1.29–2.93	0.002
	2	2.79	1.51–4.89	0.001
Increasing calprotectin day 0–day 7	1	2.46	1.11–5.46	0.027
	2	2.53	1.07–6.10	0.036

Binary logistic regression model

Adjustment models: Model 1: unadjusted. Model 2: adjusted for age, sex, BMI, hypertension, smoking and creatinine

*GOSE* Glasgow Outcome Scale Extended

^a^Odds ratio (OR) expressed per 1 Standard deviation (SD) increase in calprotectin

^b^OR for outcomes in patients with increasing calprotectin levels from day 0 to day 7

In the unadjusted model, admission and day-7 calprotectin were associated with IMV and CRRT. In the adjusted model, admission calprotectin remained significantly associated with IMV, and day-7 calprotectin was associated with CRRT (Table 4). Further, patients with increasing plasma calprotectin from ICU admission to day 7 had 2–2.5 times higher odds for mortality, unfavourable functional outcome, IMV and CRRT (Tables 2, 3 and 4).

**Table 4:** Associations between plasma calprotectin levels and the use of mechanical ventilation or renal replacement therapy

	Model	IMV			CRRT		
		OR^ab^	CI	*p*	OR^ab^	CI	*p*
Calprotectin day 0	1	1.72	1.39–2.12	< 0.001	1.02	0.80–1.32	0.86
	2	1.73	1.39–2.16	< 0.001	0.99	0.76–1.29	0.92
Calprotectin day 7	1	1.93	1.47–2.53	< 0.001	1.51	1.10–2.07	0.012
	2	1.22	0.78–1.90	0.39	1.58	1.04–2.42	0.034
Increasing calprotectin day 0–day 7	1	2.28	1.14–4.56	0.019	2.05	1.08–3.88	0.028
	2	2.23	1.10–4.53	0.027	2.07	1.07–4.00	0.031

Adjustment models: Model 1: unadjusted. Model 2: adjusted for age, sex, BMI, hypertension, smoking and creatinine

*GOSE* Glasgow Outcome Scale Extended

^a^Odds ratio (OR) expressed per 1 Standard deviation (SD) increase in calprotectin

^b^OR for outcomes in patients with increasing calprotectin levels from day 0 to day 7

In Cox proportional hazard regression analyses using the same adjusted models, we found independent associations between calprotectin levels and 12-month mortality, with an HR of 1.29 (95% CI 1.10–1.51), *p* = 0.001 for admission calprotectin, an HR of 1.93 (95% CI 1.59–2.35), *p* < 0.001 for day 7 calprotectin and a HR of 1.78 (95% CI 1.22–2.60), *p* = 0.003 for increasing calprotectin between admission and day 7 (Supplementary Table 3).

### Calprotectin as a mortality predictor

We performed a multivariable logistic regression analysis with backward factor selection, which identified age and calprotectin, both at admission and on day 7, as the strongest independent predictors of 12-month mortality (*p* < 0.001) (Supplementary Table 4). In ROC curve analyses, the model based on admission calprotectin alone had an AUC of 0.60 (*p* < 0.001). When age and admission calprotectin were combined, the AUC increased to 0.80 (*p* < 0.001). However, adding admission calprotectin did not improve the discriminative ability for 12-month mortality of the model based on age alone (*p* = 0.063) (Supplementary Table 5).

**Fig. 2:**
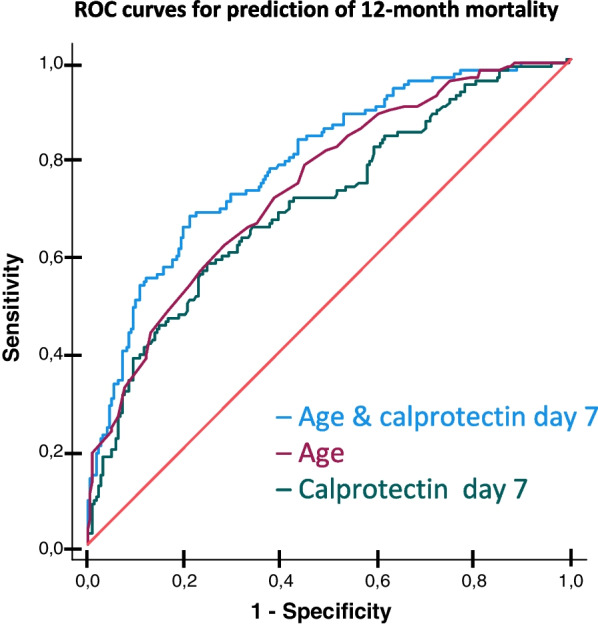
ROC curves for models including age and day 7 calprotectin as predictors of 12-month mortality. ROC curves displaying the discriminative ability of age, day 7 calprotectin, and a combined model that includes age and day 7 calprotectin to predict 12-month mortality in patients alive at 7 days (*n* = 356)

The ROC model for 12-month mortality prediction based on day 7 calprotectin had an AUC of 0.70 (*p* < 0.001), and the model based on age alone had an AUC of 0.74 (*p* < 0.001). Adding day 7 calprotectin significantly improved the discriminative ability of age to predict 12-month mortality, leading to an AUC of 0.79 for the combined model (*p* = 0.004 compared to age alone) (Fig. 2). The optimal balance between sensitivity and specificity was achieved at a day 7 calprotectin cut-off value of 5.63 mg/L (Supplementary Table 5).

### Calprotectin as a predictor of functional outcome

**Fig. 3:**
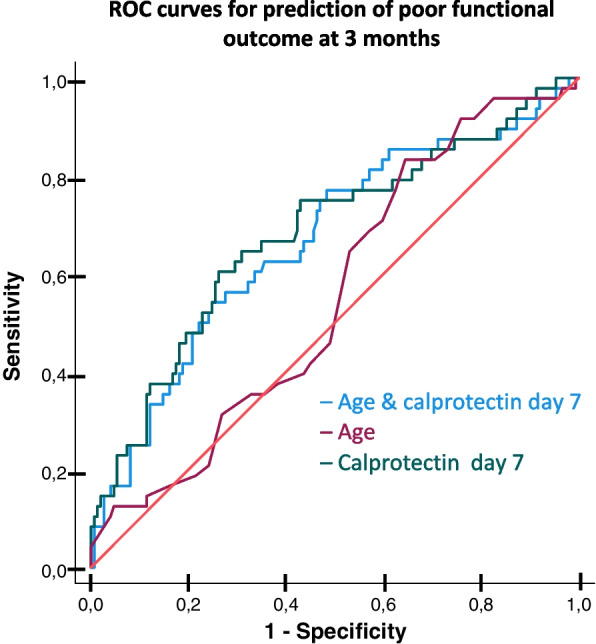
ROC curves for models including age and day 7 calprotectin as predictors of unfavourable functional outcome at 3 months. ROC curves displaying the discriminative ability of age, day 7 calprotectin, and a combined model that includes age and day 7 calprotectin to predict unfavourable functional outcome (GOSE < 5) at 3 months in patients who attended the follow-up evaluation (*n* = 264)

The ROC curve analyses revealed no discriminative ability of age, admission calprotectin, or a combination of both, to predict unfavourable functional outcome at 3 months. For the patients with available day 7 calprotectin values (*n* = 356), we found that age had no discriminative ability for unfavourable GOSE (< 5) at 3 months. The model based on day 7 calprotectin had an AUC of 0.67 (*p* < 0.001), and the combined day 7 calprotectin and age model had an AUC of 0.68 (*p* < 0.001). Adding age to calprotectin did not improve the discriminative ability of calprotectin to predict an unfavourable 3-month functional outcome (*p* = 0.57) (Fig. 3). Prediction models for unfavourable functional outcomes at 12 months could not be adequately fitted due to the small percentage of patients with a GOSE score < 5 at this time point. The cut-off value for optimal sensitivity and specificity balance of day 7 calprotectin and GOSE < 5 was 4.76 mg/L (Supplementary Table 5).

## Discussion

The main findings of our study are that increasing calprotectin levels from ICU admission to day 7 are independently associated with higher 12-month mortality, unfavourable functional outcome at 3 months, as well as IMV and CRRT use. In addition, admission calprotectin was independently associated with 12-month mortality and IMV need, while day 7 calprotectin was associated with 12-month mortality, unfavourable functional outcome at 3 months and CRRT use. Age is known to predict mortality in critical COVID-19 [18]. We found that adding day 7 plasma calprotectin to age improved the ability to predict mortality in our cohort. Furthermore, day 7 calprotectin alone was an acceptable predictor of an unfavourable functional outcome at 3 months.

Increased production of inflammatory cytokines is an essential response of the innate and adaptive immune systems to infections. However, excessive local and systemic inflammation may lead to extensive tissue damage, cytokine storm and eventually death. In COVID-19, a significant influx of neutrophils was observed in highly vascularised organs such as the lungs and kidneys [3]. The aggressive neutrophil recruitment and activation play a central role in the acute inflammatory flares leading to organ failure. One meta-analysis concluded that an elevated neutrophil-to-lymphocyte ratio is linked to disease severity and adverse outcomes in COVID-19 patients [28]. As plasma calprotectin has previously been proposed as a biomarker of neutrophil activity, high calprotectin values during critical disease may reflect excessive and sustained local and systemic neutrophil activation, leading to adverse outcomes [2, 29].

In a mouse model, SARS-CoV-2 induced aberrant activation of immature neutrophils and high calprotectin production, causing innate immune system dysregulation. Importantly, treatment with the specific calprotectin inhibitor paquinimod, which prevents the binding of calprotectin to its receptors, resulted in almost 100% survival in the lethal SARS-CoV-2 infection model by reducing the production and activation of abnormal neutrophils [30]. These findings support the hypothesis that activated neutrophils and calprotectin play direct pathogenic roles in SARS-CoV-2-induced disease and might be targeted to modulate the exaggerated immune response. In further support of this hypothesis, it has previously been shown that calprotectin blockade potently reduces systemic inflammation and mitigates cardiac failure in endotoxin-induced sepsis [31].

Previous studies have demonstrated associations between plasma calprotectin levels and COVID-19 severity during hospitalisation. Silvin et al*.* have shown that critical COVID-19 patients requiring admission to an ICU had dramatically higher plasma calprotectin levels compared to patients with mild and moderately severe disease [2]. A meta-analysis confirmed that calprotectin levels were significantly higher in COVID-19 patients who developed severe disease [13]. Moreover, increasing serial calprotectin values in COVID-19 patients admitted to a standard care unit were found to identify patients at high risk for ICU admission or death [32]. To our knowledge, the only previous study to evaluate the prognostic ability of serial calprotectin measurements in an ICU setting found that calprotectin progressively increased in patients who died in the hospital. This was, however, a small cohort study of 66 COVID-19 patients requiring ICU care [14].

In addition to these previous findings, the results of our study support the use of serial calprotectin measurements to monitor disease progression and prognosis in critically ill COVID-19 patients to aid clinical decision-making. A decrease in calprotectin levels and signs of clinical improvement may lead to earlier transfer to lower-tier units, freeing up resources for patients needing intensive care. Conversely, increasing calprotectin levels may prompt more aggressive treatment options, such as adding immunomodulatory therapies or targeted calprotectin blockers. Moreover, our data suggest that ICU survivors with high plasma calprotectin during the ICU stay might require additional rehabilitation support.

The strengths of our study include the prospective, multicentre design and the sizeable patient cohort with critical disease. Other strengths are the serial calprotectin measurements and detailed information on functional outcomes of survivors at the 3- and 12-month follow-up. Due to the very low number of patients with GOSE < 5 at 12 months, the statistical analyses generated broad confidence intervals, which is why we refrained from using the 12-month GOSE data in the regression models. Another limitation of the study is the lack of accurate information on the number of days passed from symptom onset and the lack of calprotectin measurements at hospital admission. Consequently, direct comparisons with previous studies on unselected cohorts of COVID-19 patients may be difficult. The conclusions of our study are, however, applicable to severely ill patients regardless of symptom duration and calprotectin levels prior to ICU admission. As measured by our particle-enhanced turbidimetric assay, the absolute calprotectin values cannot be directly compared to those measured in other studies using an ELISA method. Therefore, the absolute concentration thresholds cannot be directly extrapolated. Several analytical methods for measuring plasma and serum calprotectin generate different absolute values [33–35]. The current study found that dynamic calprotectin measurements had more robust associations with all the considered outcomes than single calprotectin values. Using calprotectin dynamics eliminates the need for an absolute threshold and can thereby be applied in any hospital, regardless of the measurement method employed in the local clinical laboratory.

## Conclusion

This is the first study to demonstrate that increasing plasma calprotectin levels are independently associated with a higher 12-month mortality rate, unfavourable functional outcome at 3 months, and organ failure in critically ill COVID-19 patients. We propose that serial calprotectin measurements and assessment of calprotectin dynamics, rather than absolute values, may be used to evaluate prognosis in this patient group. This would eliminate the need to define precise thresholds and is applicable in any hospital, independently of the measurement method of the local laboratory.

### Supplementary Information

Supplementary Material 1: Figure 1. Glasgow Outcome Scale Extended (GOSE) score at the 3-month follow-up visit. GOSE is an 8-grade ordinal scale measuring functional outcome, where GOSE 1 represents death, and GOSE 8 represents full recovery. GOSE <5 is deemed to reflect unfavourable recovery. Of 264 patients participating in the 3-month follow-up, 260 had complete data for GOSE. Only survivors are presented, and no patient scored GOSE 2 (vegetative state).

Supplementary Material 2: Figure 2. Glasgow Outcome Scale Extended (GOSE) score at the 12-month follow-up visit. GOSE is an 8-grade ordinal scale measuring functional outcome, where GOSE 1 represents death, and GOSE 8 represents full recovery. GOSE <5 is deemed to reflect unfavourable recovery. Of 217 patients participating in the 12-month follow-up, 215 had complete data for GOSE. Only survivors are presented, and no patient scored GOSE 2 (vegetative state).

Supplementary Material 3: Figure 3 Calprotectin levels measured on ICU day 7 days, grouped by the Glasgow Outcome Scale Extended (GOSE) score at the 3 months.

Supplementary Material 4.

Supplementary Material 5.

Supplementary Material 6.

Supplementary Material 7.

Supplementary Material 8.

## Data Availability

The datasets used in the current study are available from the corresponding author upon reasonable request.

## Abbreviations

AUC: Area under the curve
BMI: Body mass index
CCI: Charlson Comorbidity Index
CFS: Clinical Frailty Scale
CRP: C-reactive protein
CRRT: Continuous renal replacement therapy
GOSE: Glasgow Outcome Scale-Extended
HR: Hazard ratio
ICU: Intensive care unit
IMV: Invasive mechanical ventilation
LOS ICU: Length of stay in the ICU
NF-κB: Nuclear factor kappa-light-chain-enhancer of activated B cells
NLRP3: NOD-like receptor pyrin-domain-containing 3
OR: Odds ratio
P_a_O_2_/F_i_O_2_ (P/F): Arterial oxygen partial pressure ratio to fractional inspired oxygen
RAGE: Receptor for advanced glycation end-products
ROC: Receiver operating characteristics
SAPS 3: Simplified Acute Physiology Score 3
SARS-CoV-2: Severe acute respiratory syndrome coronavirus 2
SOFA: Sequential Organ Failure Assessment
TLR4: Toll-like receptor 4
