# Supplementary figures and images for: Increasing plasma calprotectin (S100A8/A9) is associated with 12-month mortality and unfavourable functional outcome in critically ill COVID-19 patients
Source: J Intensive Care. 2024 Jul 9;12:26. doi: 10.1186/s40560-024-00740-4 (PMC11232228; doi:10.1186/s40560-024-00740-4)

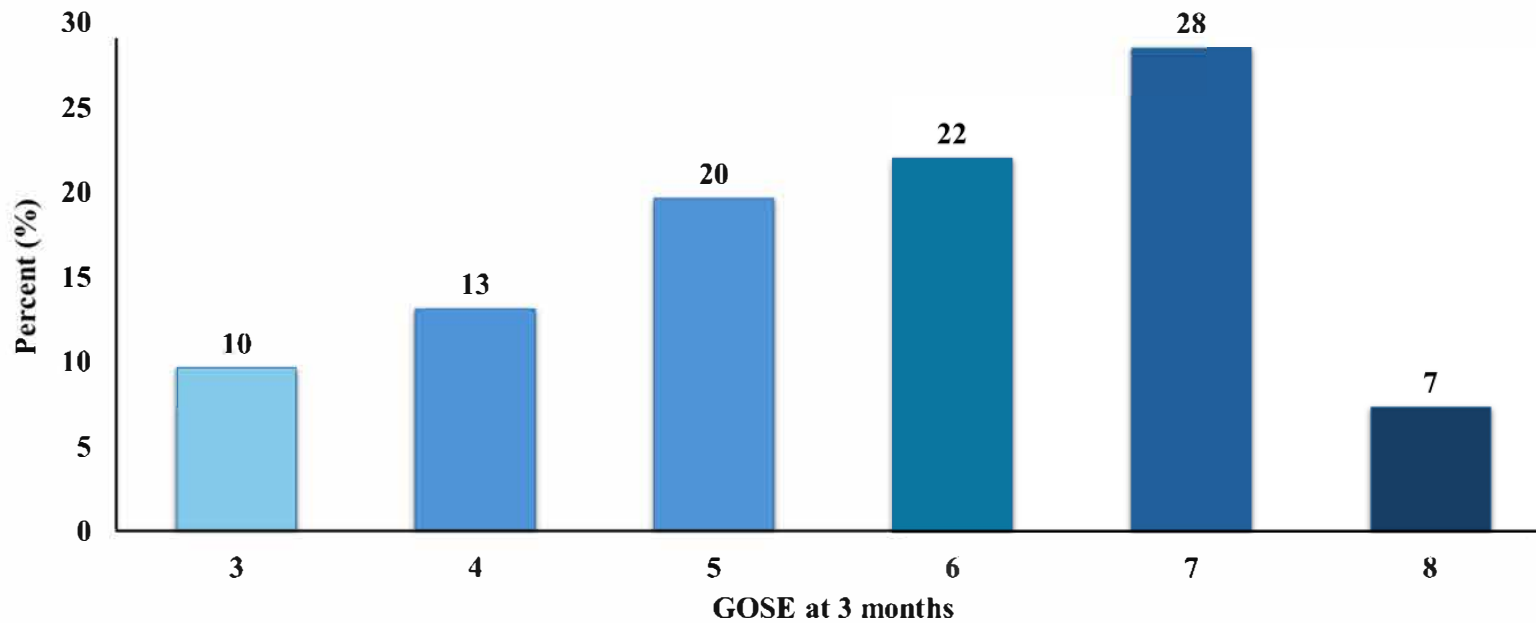

Supplement: Supplementary file 1 — Supplementary Material 1: Figure 1. Glasgow Outcome Scale Extended (GOSE) score at the 3-month follow-up visit. GOSE is an 8-grade ordinal scale measuring functional outcome, where GOSE 1 represents death, and GOSE 8 represents full recovery. GOSE <5 is deemed to reflect unfavourable recovery. Of 264 patients participating in the 3-month follow-up, 260 had complete data for GOSE. Only survivors are presented, and no patient scored GOSE 2 (vegetative state). [file 40560_2024_740_MOESM1_ESM.pdf]

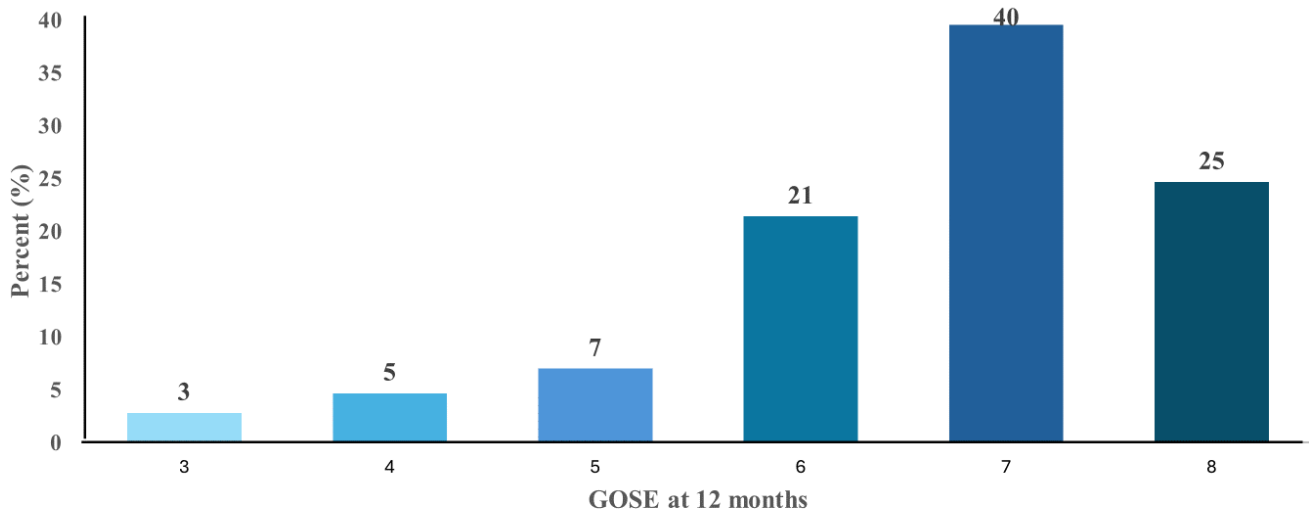

Supplement: Supplementary file 2 — Supplementary Material 2: Figure 2. Glasgow Outcome Scale Extended (GOSE) score at the 12-month follow-up visit. GOSE is an 8-grade ordinal scale measuring functional outcome, where GOSE 1 represents death, and GOSE 8 represents full recovery. GOSE <5 is deemed to reflect unfavourable recovery. Of 217 patients participating in the 12-month follow-up, 215 had complete data for GOSE. Only survivors are presented, and no patient scored GOSE 2 (vegetative state). [file 40560_2024_740_MOESM2_ESM.pdf]

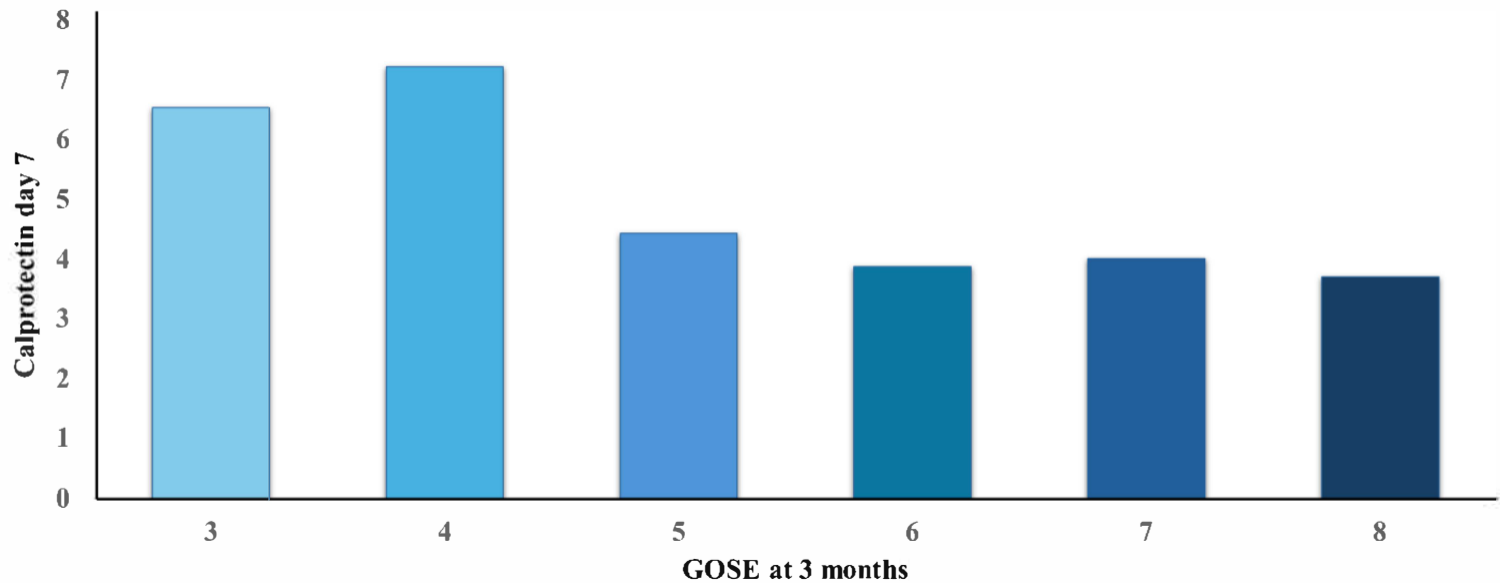

Supplement: Supplementary file 3 — Supplementary Material 3: Figure 3 Calprotectin levels measured on ICU day 7 days, grouped by the Glasgow Outcome Scale Extended (GOSE) score at the 3 months. [file 40560_2024_740_MOESM3_ESM.pdf]
