# Supplementary material for: Increasing plasma calprotectin (S100A8/A9) is associated with 12-month mortality and unfavourable functional outcome in critically ill COVID-19 patients
Source: J Intensive Care. 2024 Jul 9;12:26. doi: 10.1186/s40560-024-00740-4 (PMC11232228; doi:10.1186/s40560-024-00740-4)
Supplement: Supplementary file 4 — Supplementary Material 4. [file 40560_2024_740_MOESM4_ESM.docx]

**Supplementary Table 1. Calprotectin levels in patients with unfavourable vs favourable functional outcome at 3 months**

|  | GOSE < 5 | GOSE ≥ 5 | p-value |
| --- | --- | --- | --- |
| Calprotectin day 0 (mg/L) | N=56 | N=195 |  |
|  | 7.62 [3.44-12.9] | 6.70 [4.23-10.8] | 0.59 |
| Calprotectin day 7 (mg/L) | N =48 | N=149 |  |
|  | 5.04 [3.24-7.68] | 3.13 [1.73-4.78] | <0.001 |

*Mean and IQR of calprotectin levels on admission and on day 7 divided by patients with unfavourable functional outcome vs good functional outcome (GOSE<5 vs GOSE* ≥ 5*) at 3 months.*

|  | Decreasing calprotectin N=158 | Increasing calprotectin  N=33 | p-value |
| --- | --- | --- | --- |
| GOSE ≥ 5 | 79% | 61% | 0.024 |
| GOSE < 5 | 21% | 39% |  |

*Percentage of patients with unfavourable functional outcome vs good functional outcome among patients with increasing vs decreasing calprotectin levels during the first week of ICU stay.*
