# Supplementary material for: Increasing plasma calprotectin (S100A8/A9) is associated with 12-month mortality and unfavourable functional outcome in critically ill COVID-19 patients
Source: J Intensive Care. 2024 Jul 9;12:26. doi: 10.1186/s40560-024-00740-4 (PMC11232228; doi:10.1186/s40560-024-00740-4)
Supplement: Supplementary file 5 — Supplementary Material 5. [file 40560_2024_740_MOESM5_ESM.docx]

**Supplementary Table 2.** **Binary regression analyses of the associations between day 7 calprotectin and outcomes, excluding patients on CRRT**

|  | Model | IMV | | | 12-month mortality | | | GOSE <5 at 3 months | | |
| --- | --- | --- | --- | --- | --- | --- | --- | --- | --- | --- |
| Calprotectin day 7 |  | *OR^a^* | *CI* | *p* | *OR^a^* | *CI* | *p* | *OR^a^* | *CI* | *p* |
|  | 1 | 2.18 | 1.60-2.97 | <0.001 | 2.36 | 1.75-3.18 | <0.001 | 2.09 | 1.29-3.40 | 0.003 |
|  | 2 | 1.33 | 0.78-2.29 | 0.30 | 2.24 | 1.56-3.22 | <0.001 | 2.91 | 1.44-5.91 | 0.003 |

*^a^ Odds ratio (OR) expressed per 1 Standard deviation (SD) increase in calprotectin.*

*Adjustment models:*

*Model 1: Unadjusted*

*Model 2: Adjusted for age, sex, BMI, hypertension, smoking and creatinine.*

*IMV, invasive mechanical ventilation; GOSE, Glasgow Outcome Scale Extended.*
