# Supplementary material for: Increasing plasma calprotectin (S100A8/A9) is associated with 12-month mortality and unfavourable functional outcome in critically ill COVID-19 patients
Source: J Intensive Care. 2024 Jul 9;12:26. doi: 10.1186/s40560-024-00740-4 (PMC11232228; doi:10.1186/s40560-024-00740-4)
Supplement: Supplementary file 6 — Supplementary Material 6. [file 40560_2024_740_MOESM6_ESM.docx]

**Supplementary Table 3. Cox proportional regression analyses of the associations between plasma calprotectin and mortality**

|  |  | 12-month mortality | | |
| --- | --- | --- | --- | --- |
|  | *Model* | *HR^a,b^* | *CI* | *p* |
| Calprotectin day 0 | 1 (n=477) | 1.27 | 1.09-1.48 | 0.001 |
|  | 2 (n=451) | 1.29 | 1.10-1.51 | 0.001 |
| Calprotectin day 7 | 1 (n=356) | 1.92 | 1.60-2.32 | <0.001 |
|  | 2 (n=273) | 1.93 | 1.59-2.35 | <0.001 |
| Increasing calprotectin | 1 (n=346) | 1.82 | 1.25-2.63 | 0.002 |
|  | 2 (n=332) | 1.78 | 1.22-2.60 | 0.003 |
| Calprotectin day 7 in patients  without CRRT ^b^ | 1 (n=304) | 2.01 | 1.64-2.47 | <0.001 |
|  | 2 (n=292) | 1.98 | 1.61-2.44 | <0.001 |

*^a^ Hazard ratio (HR) per 1 Standard deviation (SD) increase in calprotectin.*

*^b^ Calprotectin on day 7 analysed in the subset of patients without continuous renal replacement therapy (CRRT)*

*Adjustment models:*

*Model 1: Unadjusted*

*Model 2: Adjusted for age, sex, BMI, hypertension, smoking and creatinine.*
