# Supplementary material for: Increasing plasma calprotectin (S100A8/A9) is associated with 12-month mortality and unfavourable functional outcome in critically ill COVID-19 patients
Source: J Intensive Care. 2024 Jul 9;12:26. doi: 10.1186/s40560-024-00740-4 (PMC11232228; doi:10.1186/s40560-024-00740-4)
Supplement: Supplementary file 7 — Supplementary Material 7. [file 40560_2024_740_MOESM7_ESM.docx]

**Supplementary Table 4. Binary regression analysis with backward variable selection for 12-month mortality**

|  | OR ^a^ | CI | p |
| --- | --- | --- | --- |
| Calprotectin day 0 | 1.51 | 1.21-1.90 | <0.001 |
| Age | 4.27 | 2.93-6.22 | <0.001 |
| Smoker | 1.63 | 1.05-2.53 | 0.029 |
| Creatinine | 1.34 | 1.07-1.67 | 0.009 |
|  |  |  |  |
| Calprotectin day 7 | 2.02 | 1.45-2.80 | <0.001 |
| Age | 2.69 | 1.76-4.13 | <0.001 |
| Smoker | 1.86 | 1.08-3.20 | 0.025 |

*^a^ Odds ratio (OR) expressed per 1 Standard deviation (SD) increase in calprotectin, age and creatinine.*

*Binary logistic regression analyses with backward variable selection including 12-month mortality as outcome and calprotectin (day 0 and day 7 included in separate models), age, sex, BMI, hypertension, smoking and creatinine.*
