# Supplementary material for: Increasing plasma calprotectin (S100A8/A9) is associated with 12-month mortality and unfavourable functional outcome in critically ill COVID-19 patients
Source: J Intensive Care. 2024 Jul 9;12:26. doi: 10.1186/s40560-024-00740-4 (PMC11232228; doi:10.1186/s40560-024-00740-4)
Supplement: Supplementary file 8 — Supplementary Material 8. [file 40560_2024_740_MOESM8_ESM.docx]

**Supplementary Table 5. AUC for the different prediction models**

| **Calprotectin day 0 and 12-month mortality (n=498)** | AUC | 95% C.I. | p* |
| --- | --- | --- | --- |
| Age | *0.790* | *0.74-0.83* | <0.001 |
| Calprotectin day 0 | *0.600* | *0.54-0.65* | <0.001 |
| Age & Calprotectin day 0 | *0.800* | *0.76-0.84* | <0.001 |

| **Calprotectin day 7 and 12-month mortality (n=356)** | AUC | 95% C.I. | p* |
| --- | --- | --- | --- |
| Age | 0.74 | 0.69-0.79 | <0.001 |
| Calprotectin day 7 | 0.70 | 0.65-0.76 | <0.001 |
| Age & Calprotectin day 7 | 0.79 | 0.74-0.84 | <0.001 |

| **Calprotectin day 0 and GOSE < 5 at 3 months (n= 260)** | AUC | CI | p |
| --- | --- | --- | --- |
| Age | 0.590 | 0.51-0.67 | 0.027 |
| Calprotectin day 0 | 0.530 | 0.43-0.62 | 0.600 |
| Age & Calprotectin day 0 | 0.590 | 0.51-0.67 | 0.026 |

| **Calprotectin day 7 and GOSE < 5 at 3 months (n=260)** | AUC | 95% C.I. | p* |
| --- | --- | --- | --- |
| Age | 0.56 | 0.47-0.65 | 0.22 |
| Calprotectin day 7 | 0.67 | 0.58-0.76 | <0.001 |
| Age & Calprotectin day 7 | 0.68 | 0.59-0.77 | <0.001 |

*AUC and 95% CI for three different ROC models predicting 12-month mortality and GOSE <5 at 3 months. The first model is based on age, the second is based on calprotectin levels (on admission or on day 7), and the third combines age and calprotectin.*

** p-value of the comparison with the null hypothesis.*

**Comparison of the three prediction models with the DeLongs test**

| **Calprotectin day 0 and 12-month mortality** | p |
| --- | --- |
| Age vs Calprotectin day 0 | <0.001 |
| Age & Calprotectin day 0 vs Calprotectin day 0 | <0.001 |
| Age & Calprotectin day 0 vs Age | 0.063 |

| **Calprotectin day 7 and 12-month mortality** | p |
| --- | --- |
| Age vs Calprotectin day 7 | 0.36 |
| Age & Calprotectin day 7 vs Calprotectin day 7 | 0.003 |
| Age & Calprotectin day 7 vs Age | 0.004 |

| **Calprotectin day 0 and GOSE <5 at 3 months** | p |
| --- | --- |
| Age vs - Calprotectin day 0 | 0.320 |
| Age & Calprotectin day 0 vs Calprotectin day 0 | 0.280 |
| Age& Calprotectin day 0 vs Age | 0.920 |

| **Calprotectin day 7 and GOSE <5 at 3 months** | p |
| --- | --- |
| Age vs Calprotectin day 7 | 0.098 |
| Age & Calprotectin day 7 vs Calprotectin day 7 | 0.57 |
| Age & Calprotectin day 7 vs Age | 0.015 |

*Calprotectin on ICU admission and day 7 vs. 12-month mortality and functional outcome. The models include age, calprotectin, and a combination of age and calprotectin. The three prediction models have been compared with the DeLongs test.*
